# Antimicrobial Effect of Oregano Essential Oil in Na-Alginate Edible Films for Shelf-Life Extension and Safety of Feta Cheese

**DOI:** 10.3390/pathogens15010065

**Published:** 2026-01-08

**Authors:** Angeliki Doukaki, Aikaterini Frantzi, Stamatina Xenou, Fotoula Schoina, Georgia Katsimperi, George-John Nychas, Nikos Chorianopoulos

**Affiliations:** Laboratory of Microbiology and Biotechnology of Foods, Department of Food Science and Human Nutrition, School of Food and Nutritional Sciences, Agricultural University of Athens, Iera Odos 75, 11855 Athens, Greece; adoukaki@aua.gr (A.D.); kathrinfran14@gmail.com (A.F.); stamatinaxenou@aua.gr (S.X.); fschoina@aua.gr (F.S.); stud218036@aua.gr (G.K.); gjn@aua.gr (G.-J.N.)

**Keywords:** feta cheese, edible films, oregano essential oil, *Escherichia coli*, *Listeria monocytogenes*, multispectral imaging

## Abstract

The use of natural antimicrobials and advanced sensor technologies is increasingly explored to improve the safety and quality of dairy products like cheese. The current work evaluated the effect of sodium alginate edible films enriched with oregano essential oil (EO) on the microbial spoilage of Feta cheese and the fate of *Escherichia coli* O157:H7 and *Listeria monocytogenes* during storage. Samples were inoculated with approximately a 4 log CFU/g of pathogens and subsequently wrapped with edible films containing EO or left without, serving as controls. Samples were stored under aerobic and vacuum conditions at 4 and 12 °C. Microbiological analyses, pH, and sensory attributes were monitored during storage, while multispectral imaging (MSI) devices were used for rapid, non-invasive quality assessment. EO films moderately suppressed spoilage and pathogen survival, particularly under aerobic conditions. The MSI spectral data coupled with machine learning models provided reasonable results for the estimation of yeast and mould populations, with the best models coming from aerobic conditions, from benchtop-MSI data, with R^2^ = 0.726 and RMSE = 0.426 from the Neural Networks model, and R^2^ = 0.661 and RMSE = 0.696 from the LARS model. The results highlight the combined potential of natural antimicrobial films and MSI-based sensors for extending Feta cheese shelf life and enabling rapid, non-destructive monitoring, respectively.

## 1. Introduction

Feta cheese is a white-brined traditional Greek cheese widely consumed both nationally and internationally, representing nearly 90% of the total national cheese production in Greece [1]. Due to its long-standing reputation and strong cultural identity, it holds a prominent position in domestic and export markets and ranks first among Greek cheese products in global sales [2]. Since 2002, Feta cheese has been documented as a Protected Designation of Origin (PDO) product under Commission Regulation (EC) No. 1820/2002 [3]. It is manufactured from sheep milk or a mixture of sheep milk with up to a maximum of 30% goat milk [4]. In industrial large-scale production, it is produced by pasteurised milk with the use of lactic acid bacteria (LAB) starter cultures to ensure controlled acidification of the milk [5]. *Lactococcus lactis, Lactobacillus delbrueckii* subsp. *bulgaricus, Streptococcus thermophilus*, and *Leuconostoc lactis* are indeed among the most widely used lactic acid bacteria (LAB) starter cultures [6,7]. During cheese production and maturation, both starter LAB (SLAB), which drive acidification, and non-starter LAB (NSLAB), which contribute to lipolysis and proteolysis and hence to flavour and texture development, play a dynamic role in forming the final organoleptic and physicochemical characteristics of Feta [8]. 

Feta is highly susceptible to yeast and mould spoilage, which may originate from the processing equipment, air, raw materials, brine tanks, wooden ripening racks, and employees [9]. Their proliferation causes undesirable sensory changes to cheese, leading to a decrease in shelf life and compromising product quality [10,11]. Despite being a generally safe product, cheese has been involved in foodborne outbreaks and recalls [12]. Several compositional factors of cheese, such as relatively high protein, fat, moisture, and micronutrient content, further support the growth and persistence of these microorganisms in the right conditions [13]. In addition to spoilage microorganisms, Feta may also be contaminated with foodborne pathogens such as *Escherichia coli* O157:H7, *Listeria monocytogenes*, *Salmonella* spp., *Yersinia enterocolitica*, and *Staphylococcus aureus*, especially when hygiene conditions are inadequate [14,15].

More specifically, *Listeria monocytogenes* is of major concern in cheese production due to its ability to survive and grow under conditions that inhibit most pathogens, including refrigeration temperatures, a range of pH values, and salt levels [16,17]. The pasteurisation of milk is the only safety step applied before the consumption of cheese; however, it does not secure the lack of *L. monocytogenes* in case of improper techniques [18]. In addition, post-pasteurisation cross-contamination is possible from improper handling [19,20]. On the other hand, *Escherichia coli* is a heat-sensitive pathogen and typically eliminated through proper pasteurisation; however, it remains a pathogen found in dairy products [21,22]. Some studies have reported the presence of Shiga toxin-producing *E. coli* (STEC) in cheeses made from pasteurised milk, highlighting that inadequate pasteurisation or post-pasteurisation cross-contamination during handling and processing can reintroduce the pathogen [23,24]. *E. coli* O157:H7 can also persist under environments typically inhibitory to many bacteria. Although its optimal growth occurs near neutral pH, some strains can survive refrigeration temperatures or prolonged exposure to acidic environments (pH 3.0–4.0), specifically suggesting that the acidification driven by LAB during cheese fermentation may not be sufficient to ensure its elimination [25,26,27].

Given the susceptibility of Feta cheese and other white-brined cheeses to spoilage microorganisms and post-processing contamination by foodborne pathogens, there is a need for improved preservation strategies that can reinforce microbial safety while maintaining product quality. In parallel, increasing environmental concerns and regulatory restrictions related to regular cheese packaging materials, such as polyethylene, polyamide, and polypropylene, have intensified interest in ‘green’ packaging alternatives. These petroleum-based materials are non-biodegradable and non-edible, and their use may raise concerns related to material migration into the cheese matrix [13]. In this context, active packaging has gained considerable attention, as it incorporates components that can deliberately release or absorb substances to delay deterioration, inhibit microbial growth, or maintain quality [28,29]. A subset of these technologies involves edible films and coatings, which act as protective barriers against moisture loss or microbial contamination [30,31]. Biopolymers derived from natural sources have appeared as capable candidates due to their environmental compatibility and suitability for food contact applications [32]. Among them, sodium alginate (Na-alginate), a polysaccharide extracted from brown seaweed, has attracted considerable attention owing to its non-toxicity, biodegradability, low price, and ability to form edible films and coatings [33,34].

To improve the functional performance, the physicochemical, mechanical, and microbiological properties of such biopolymeric packaging materials, active agents are often incorporated into these matrices, including plant-derived essential oils [35,36]. Essential oils such as oregano, thyme, and rosemary contain phenolic compounds (e.g., carvacrol, thymol) that are effective against spoilage microorganisms and key foodborne pathogens [37,38]. Oregano EO is considered one of the most potent natural antimicrobials, exhibiting robust activity against bacteria and fungi. Even though oregano EO is deemed as GRAS (generally recognised as safe), due to its intense aroma and flavour, its application directly to foods may be limited [37]. Therefore, its incorporation into edible films for cheese preservation has been proposed as an effective strategy to control microbial growth while minimising sensory impact. Previous studies have demonstrated that EOs incorporated into edible films may moderate microbial proliferation and delay the spoilage of dairy products [39,40,41].

To complement such preservation strategies, rapid and non-invasive methods for monitoring microbial quality have also gained interest. Traditional methods for assessing food quality are often destructive to the samples, time-consuming, and require trained personnel, providing retrospective information [42]. Recent advantages involve a wide range of spectroscopic and imaging technologies [43,44]. Under the imaging-based methods, multispectral imaging (MSI), though less explored than hyperspectral imaging (HSI), has been a capable approach for non-invasive assessment of quality in various food commodities [45,46,47]. MSI acquires spatial information across a limited set of discrete wavelengths, providing valuable insights into the ‘surface chemistry’ of food products [48]. In combination with machine learning models, such techniques are a promising tool for quality control within the food supply chain.

Based on the above, the efficacy of both edible films and essential oils can vary depending on the food matrix, storage temperature, and packaging, factors relevant for dairy products such as Feta cheese. Hence, further research is required to assess their practical performance under realistic storage conditions. In the present study, the effect of oregano EO added to sodium alginate edible films on the microbiological and sensory properties of Feta stored under aerobic and vacuum conditions at 4 °C and 12 °C was assessed. The applied treatment was also tested for the suppression of two known pathogens found in dairy, *Listeria monocytogenes* and *Escherichia coli* O157:H7. The feasibility of machine learning models combined with multispectral imaging (MSI) sensors was evaluated to predict the microbial quality of Feta cheese and further enhance the research aim.

## 2. Materials and Methods

### 2.1. Experimental Design

The Feta cheese samples were inoculated with a 3-strain cocktail of either *Listeria monocytogenes* or *Escherichia coli* O157:H7 and then wrapped in Na-alginate edible films, with or without oregano essential oil (EO), to study their effect on shelf-life extension and inhibition of the pathogens. During storage, microbiological, pH, and sensory analyses were conducted, in parallel with the use of MSI sensors.

### 2.2. Microbial Cultures

In the present study, the *L. monocytogenes* strains used were FMCC-B128, FMCC-B133 (isolated from soft cheese), and FMCC-B160 (isolated from a food processing plant). For *E. coli*, the strains used were FMCC-B15 (isolated from human faeces/Serotype O157:H7/Produces Vero cytotoxins VT1 and VT2), FMCC-B16, and FMCC-B18 (Serotype O157:H7/Vero cytotoxins negative). All FMCC strains belong to the culture collection of the Agricultural University of Athens, Greece. Monocultures of the strains were recovered from pure cultures stored in −80 °C supplemented with 20% glycerol. *L. monocytogenes* strains were inoculated in Brain Heart Infusion Broth (BHI Broth, NCM0016B, Neogen, Lansing, MI, USA) and *E. coli* strains in Tryptone Soy Broth (TSB, NCM0019A, Neogen, Kansas, MO, USA), following incubation at 37 °C for 24 h. The subculture of each strain was prepared in 10 mL of fresh broth and incubated at 37 °C for 18 h. To obtain the biomass of the cells (pellet), centrifugation (6000× rpm, 10 min, 4 °C) of the overnight cultures was performed, and the pellet was then washed twice with sterile ¼ strength Ringer’s solution (NCM0191K, Neogen) and resuspended in 10 mL of Ringer’s solution. To confirm the inoculum size of each strain, serial dilutions were prepared and inoculated on *Listeria* Palcam Agar (Ref. 4016042, Biolife, Italiana S.r.l, Milano, Italy) with Palcam selective supplement (*Listeria* Palcam Antimicrobic Supplement, Ref. 4240042, Biolife), incubated at 37 °C for 48 h for *L. monocytogenes* and Tryptone Bile Glucuronide Agar (TBX, 4021562, Biolife, Milano, Italy), incubated at 44 °C for 24 h, for *E. coli*. Final inoculum was prepared by mixing the biomass of each pathogen strain to accomplish a total population of approximately 4 log CFU/g.

### 2.3. Na-Alginate Edible Films

Formulation of the Na-alginate edible films was accomplished as previously defined by Pavli et al. [49]. The Na-alginate edible films were prepared at a final concentration of 2% *w/v*, with and without the addition of oregano EO. In the case with the addition of oregano EO, a concentration of 0.5% *v/v* of the EO (Ecopharm Hellas S.A., Macedonia, Greece) was added under constant agitation at the forming solution step. Approximately 20 g of the final Na-alginate solution (with or without EO) was poured into square Petri dishes, used as a matrix to form the films, and then allowed to dry for 12 h inside a laminar flow cabinet at ambient temperature. To detach the films from the Petri dishes, 20 mL aliquots of 2% *w*/*v* CaCl_2_ were added for 1 min, and after the films were dry, they were stored at 4 °C until use.

### 2.4. Inoculation and Preparation of Cheese Samples

Feta cheese (5% fat) from Family Farm industry (Almyros, Volos, Greece) had been ripened for 90 days in 20 kg containers (4% *w/v* brine) and was transported to the laboratory before being released onto the market, with a shelf life of 90 days in plastic containers. 

The cheese was first sliced into 180 g pieces under aseptic conditions before further processing. Half of the samples (inoculated) were transferred into a laminar flow cabinet, where they were inoculated with 180 μL of each three-strain pathogen cocktails and spread evenly across the cheese’s entire surface to achieve a 4 log CFU/g population in the final product. The rest of the samples (non-inoculated) served as the control. Slices from both cases (non-inoculated, inoculated) were then cut into 40 g portions, wrapped in Na-alginate edible films (no EO, with EO), and packaged under aerobic or vacuum conditions using sterile pouches (width 25 cm, thickness 90 μm, permeability at 20 °C, and 50% RH: 25, 90, and 6 cm^3^·m^−2^·day^−1^·bar^−1^ for CO_2_, O_2_, and N_2_, respectively, Flexopack S.A., Athens, Greece) and a HenkoVac 1900 machine (Howden Food Equipment B.V., Hertogenbosch, The Netherlands).

Samples of all cases were stored in high-precision incubators (MIR153, Sanyo Electric Co., Osaka, Japan) at 4 °C and 12 °C until spoilage. A description of the different sample cases is presented in Table 1.

### 2.5. Microbiological Analyses and pH Measurements

Microbial analyses of the Feta cheese samples were implemented during storage from day 0 up to day 31 for aerobic conditions and day 72 for vacuum, depending on different sample treatments, temperatures, and packaging conditions. During the sampling procedure, 10 g of the samples were aseptically added to sterile stomacher bags (BagLight^®^, INTERSCIENCE, Paris, France) with 90 mL of sterilised ¼ Ringer’s solution and were then homogenised using a Stomacher (Lab Blender 400, Seward Medical, London, UK) for 60 s at room temperature. The Na-alginate edible films were removed from the samples prior to analysis. Further serial decimal dilutions in the same diluent were prepared, and 0.1 or 1 mL of the appropriate dilution was inoculated using the spread-or-pour method, respectively, on the following non-selective and selective agar media: Plate Count Agar (Tryptic Glucose Yeast Agar PCA, Ref. 4021452, Biolife, Italiana S.r.l, Milano, Italy) for the enumeration of total aerobic viable counts (TVCs), incubated at 30 °C for 72 h; MRS Agar (401728S2, Biolife, Milano, Italy) overlaid with the same medium and incubated at 30 °C for 72 h for the enumeration of mesophilic lactic acid bacteria (LAB); M17 Agar (Ref: 401719S2, Biolife, Milano, Italy) overlaid with the same medium, for the enumeration of lactic streptococci, incubated at 37 °C for 48 h; Rose Bengal Chloramphenicol agar (RBC; NCM0135, Neogen, Lansing, MI, USA) for the enumeration of yeasts/moulds, supplemented with selective supplement Chloramphenicol (NCM4051-0.5, Neogen, Lansing, MI, USA), incubated at 25 °C for 48–72 h; Violet Red Bile Glucose Agar (VRBGA, 4021884, Biolife, Milano, Italy) for the enumeration of *Enterobacteriaceae* counts overlaid with the same medium and incubated at 37 °C for 18–24 h; Tryptone Bile Glucuronide Agar (TBX, 021562, Biolife, Milano, Italy) for the enumeration of *E. coli*, incubated at 44 °C for 18–24 h; and Agar *Listeria* Ottaviani Agosti (ALOA^â^, 401605, Biolife, Milano, Italy) with ALOA^â^ enrichment selective supplement (423501, Biolife, Milano, Europe) for the enumeration of *L. monocytogenes* incubated at 37 °C for 24 h. Non-inoculated samples were also examined for the absence of the target pathogens.

Following microbiological analyses, pH values of the Feta samples were measured with a digital pH metre Russell RL150 (Russell Inc., Cork, Ireland), where the glass electrode (Metrohm AG, Herisau, Switzerland) was dipped in the homogenised sample (stomacher bag).

### 2.6. Sensory Assessment

Sensory assessment of Feta cheese samples was conducted by a semi-trained panel of five laboratory staff members. All panellists provided consent prior to participation in the study. The characteristics selected were based on a former study [50]. Non-inoculated samples were examined for their total appearance, colour (typical white colour), aroma (familiar, i.e., buttery or mildly acidic, smell of the cheeses, rancid, and/or high acidic aroma of the samples), texture (firmness, chewiness, cohesiveness, and overall mouthfeel), and taste (buttery, acid, sweet, salty, bitter, or rancid). A three-class scoring scheme was applied based on previous works [51], where the first class (0–1, fresh) was linked with fresh samples; the second class (1.5–2, marginal) with semi-fresh samples (2, accepted); and the third class (2.5–3, unacceptable) with spoiled samples. The end of shelf life for a sample was defined as the point at which at least half of the sensory panel assigned a negative score for the attributes mentioned above. 

### 2.7. Multispectral Imaging Analysis

Multispectral imaging (MSI) analysis was conducted using a VideometerLab (benchtop-MSI) and a VideometerLite (portable-MSI) instrument (Videometer A/S, Herlev, Denmark). The VideometerLab instrument captures images at 18 discrete wavelengths ranging from ultraviolet to short-wave near-infrared region (405, 435, 450, 470, 505, 525, 570, 590, 630, 645, 660, 700, 850, 870, 890, 910, 940, and 970 nm). The VideometerLite system is designed to acquire images at 10 discrete wavelengths ranging from 405 to 850 nm, specifically at 405, 403, 450, 490, 515, 590, 630, 660, 690, and 850 nm. More details regarding the MSI instruments have been previously described [52,53]. The Feta cheese samples were placed on Petri dishes and then placed inside the Ulbricht sphere of the VideometerLab or manually transferred under the sphere of the portable VideometerLite. Multispectral images of the samples were then acquired. For each sample, two image replicates were taken from both sides of the Petri dish. Image segmentation was applied using the VideometerLab system software (version 2.12.39) to select the region of interest (ROI) of the samples and remove non-relevant areas (e.g., sample background, Petri dish). Canonical discriminant analysis (CDA) was employed, resulting in segmented images. The intensity of the pixels within the ROI at each specific wavelength was then calculated as an average value and standard deviation. In total, 262 samples and 262 samples were acquired from VideometerLab and VideometerLite, respectively, for aerobic storage in *L. monocytogenes* case (including C, O, CL, and L samples), 244 and 244 for aerobic storage in *E. coli* case (including C, O, CE, and E samples), 244 and 244 for vacuum in *L. monocytogenes* case (including C, O, CL, and L samples), and 224 and 221 for vacuum in *E. coli* case (including C, O, CE, and E samples). The edible films were removed before acquiring the images on both instruments.

### 2.8. Data Analysis

All experiments were performed using two independent batches of Feta samples with two replicates each. Data were converted to log CFU/g, and the average and standard deviation (SD) of log CFU/g were calculated for each sampling point. The results were analysed for statistical significance (*p* < 0.05) with analysis of variance (ANOVA), and Duncan’s multiple range test was applied to determine the significant differences among the results. Calculations were performed using the XLSTAT^®^ software (version 2023.3.1, Addinsoft, New York, NY, USA).

The ability of multispectral imaging (MSI) to predict the microbial spoilage of Feta cheese was evaluated using different combinations of preprocessing methods and machine learning regression models to identify the most accurate predictive pipeline. The MSI data from both instruments were combined with the corresponding mean log CFU/g values for the yeast and mould counts of each experimental case. To improve data quality and remove unwanted variation in the spectral signals, the preprocessing methods tested were centring and scaling to standardise the data, so all wavelengths contributed equally; standard normal variate (SNV) normalisation was also used to correct the scattering effect in the spectra, or a combination of the two. Stratified sampling was applied, where 80% of the data was used for training and internal validation of the models, and 20% for testing them. Model training employed repeated k-fold cross-validation (5 folds, 3 repetitions) to ensure robustness and reduce overfitting. The following nine machine learning regression models were evaluated: Partial Least Squares Regression (PLS-Rs), Principal Component Regression (PCR), Ridge Regression, Support Vector Regression (SVM—Linear kernel), Support Vector Regression (SVM—Radial kernel), Random Forest Regression, k-Nearest Neighbours Regression (kNN), Neural Network Regression (NNet), and Least Angle Regression (LARS). Each algorithm was tested under all preprocessing combinations. The performance of the models was assessed based on the following four metrics: R^2^ (coefficient of determination), which indicates how much of the variability in bacterial counts is explained by the model, RMSE (root mean square error), which measures the average prediction error, MAE (mean absolute error), which is the average absolute difference between predicted and observed values, and accuracy (±1 log CFU/g) for the proportion of predictions within ±1 log unit of the true bacterial count. While accuracy is not typically applied to regression models, in predictive microbiology, it can be adapted and indicate the proportion of predictions falling within the ±1 log unit of the observed value, as deviations within this range correspond to 1 dilution and are considered acceptable for practical purposes [54,55]. Data analysis was performed using the statistical language R (ver. 4.5.2) [56] in RStudio (ver. 2025.09.1+401) [57].

## 3. Results

### 3.1. Microbiological Analyses and pH

**Figure 1:**
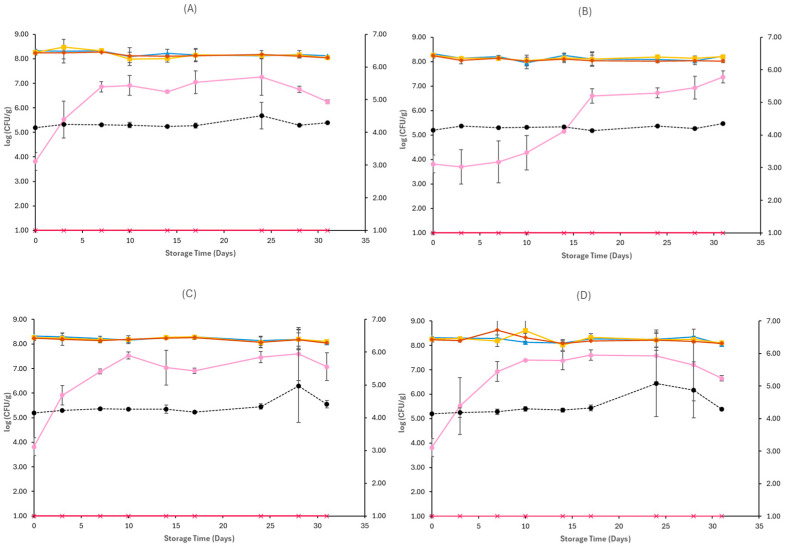
Populations (mean ± standard deviation) of TVC (*●*), LAB (*●*), Cocci/Streptococci (*●*), yeasts/moulds (*●*), and *Enterobacteriaceae* (*●*), on aerobically stored Feta cheese C samples stored at 4 °C (**A**), O samples stored at 4 °C (**B**), C samples stored at 12 °C (**C**), and O samples stored at 12 °C (**D**). pH values (●) are indicated in the secondary axis and are represented with a dotted line (…).

**Figure 2:**
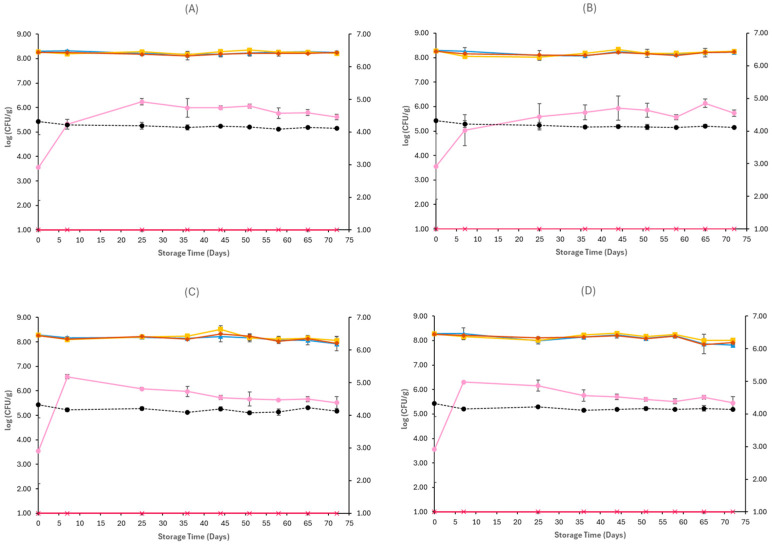
Populations (mean ± standard deviation) of TVC (*●*), LAB (*●*), Cocci/Streptococci (*●*), yeasts/moulds (*●*), and *Enterobacteriaceae* (*●*), on vacuum-stored Feta cheese C samples stored at 4 °C (**A**), O samples stored at 4 °C (**B**), C samples stored at 12 °C (**C**), and O samples stored at 12 °C (**D**). pH values (●) are indicated in the secondary axis and are represented with a dotted line (…).

**Figure 3:**
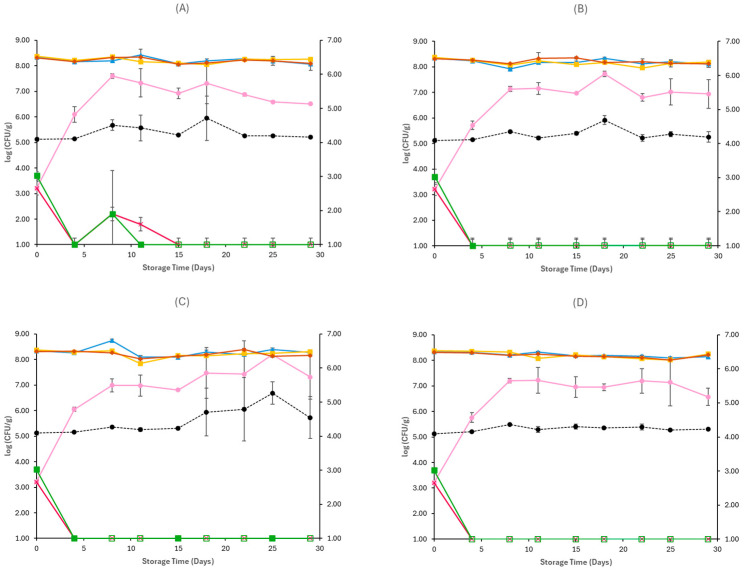
Populations (mean ± standard deviation) of TVC (*●*), LAB (*●*), Cocci/Streptococci (*●*), yeasts/moulds (*●*) and *Enterobacteriaceae* (*●*), and *E. coli* O157:H7 (*●*), on aerobically stored Feta cheese CE samples stored at 4 °C (**A**), OE samples stored at 4 °C (**B**), CE samples stored at 12 °C (**C**), and OE samples stored at 12 °C (**D**). pH values (●) are indicated in the secondary axis and are represented with a dotted line (…).

**Figure 4:**
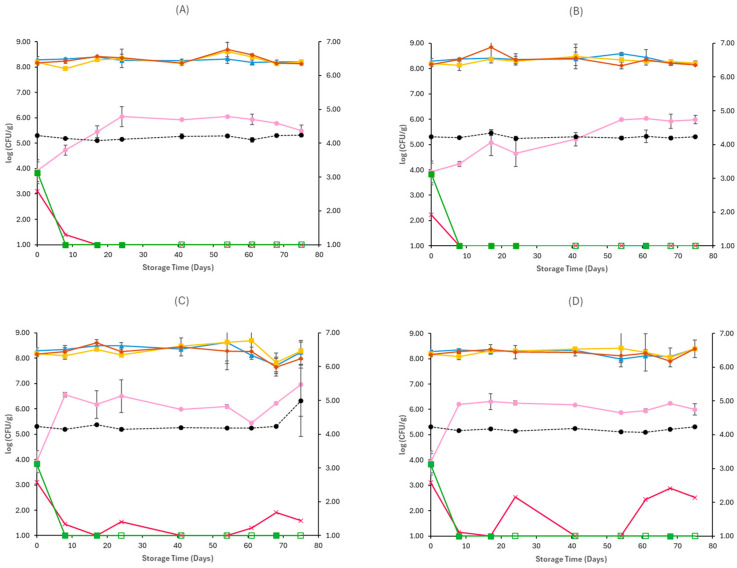
Populations (mean ± standard deviation) of TVC (*●*), LAB (*●*), Cocci/Streptococci (*●*), yeasts/moulds (*●*) and *Enterobacteriaceae* (*●*), and *E. coli* O157:H7 (*●*), on vacuum-stored Feta cheese CE samples stored at 4 °C (**A**), OE samples stored at 4 °C (**B**), CE samples stored at 12 °C (**C**), and OE samples stored at 12 °C (**D**). pH values (●) are indicated in the secondary axis and are represented with a dotted line (…).

**Figure 5:**
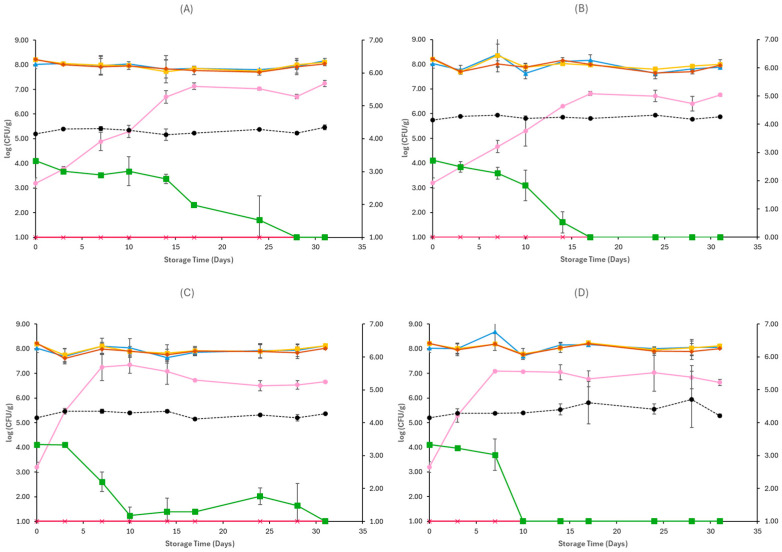
Populations (mean ± standard deviation) of TVC (*●*), LAB (*●*), Cocci/Streptococci (*●*), yeasts/moulds (*●*) and *Enterobacteriaceae* (*●*), and *L. monocytogenes* (*●*), on aerobically stored Feta cheese CL samples stored at 4 °C (**A**), OL samples stored at 4 °C (**B**), CL samples stored at 12 °C (**C**), and OL samples stored at 12 °C (**D**). pH values (●) are indicated in the secondary axis and are represented with a dotted line (…).

**Figure 6:**
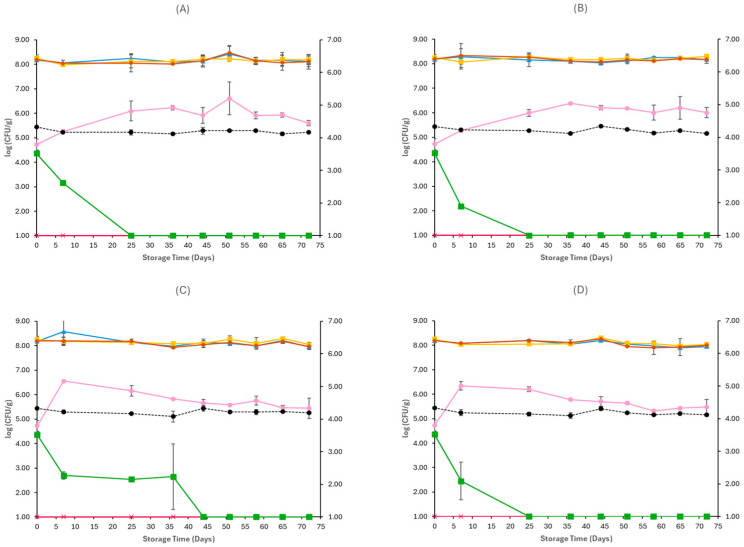
Populations (mean ± standard deviation) of TVC (*●*), LAB (*●*), Cocci/Streptococci (*●*), yeasts/moulds (*●*) and *Enterobacteriaceae* (*●*), and *L. monocytogenes* (*●*), on vacuum-stored Feta cheese CL samples stored at 4 °C (**A**), OL samples stored at 4 °C (**B**), CL samples stored at 12 °C (**C**), and OL samples stored at 12 °C (**D**). pH values (●) are indicated in the secondary axis and are represented with a dotted line (…).

Figure 1, Figure 2, Figure 3, Figure 4, Figure 5 and Figure 6 show the populations of the tested microorganisms and pH values for the different sample cases. Initial TVC of Feta cheese was 8.21 log CFU/g (±0.13), and the yeast/moulds population was 3.76 log CFU/g (±0.28). For LAB and lactic cocci/streptococci, the population was 8.20 log CFU/g (±0.15) and 8.19 log CFU/g (±0.10), respectively, and no *Enterobacteriaceae* were detected in control samples. The initial pH value was 4.31 (±0.12).

In non-inoculated samples stored under both aerobic and vacuum conditions, at 4 and 12 °C, the TVC, LAB, and lactic cocci/streptococci populations remained relatively stable throughout storage, with levels of around 8–8.5 log CFU/g at both sample cases (C, O), as seen in Figure 1 and Figure 2. At the end of storage in aerobic conditions, the yeast/mould population for C samples at 4 °C was higher compared to initial populations and achieved 6.24 log CFU/g (±0.08) and for O samples 7.37 log CFU/g (±0.24), while at 12 °C it was 7.07 log CFU/g (±0.57) for C samples and 6.65 log CFU/g (±0.11) for O samples. A delay of yeast/mould growth on O samples compared to C samples was observed during storage at 4 °C, as seen in Figure 1, indicating a moderate effect of oregano EO added to Na-alginate edible films in spoilage delay. In the case of vacuum-stored Feta cheese, the population of yeast/moulds at the end of storage at 4 °C and 12 °C for C samples was 5.62 log CFU/g (±0.11) and 5.52 log CFU/g (±0.24), respectively, while for O samples, 5.73 log CFU/g (±0.14) and 5.46 log CFU/g (±0.25), respectively. As depicted in Figure 2, no visible effect of oregano EO on spoilage was observed. For both sample cases (C and O) and storage conditions at 4 and 12 °C, no *Enterobacteriaceae* were detected. The pH values on aerobic conditions at the end of storage at 4 and 12 °C were similar across C (4.30 ± 0.01 log CFU/g, 4.41 ± 0.11 log CFU/g) and O (4.35 ± 0.01 log CFU/g, 4.29 ± 0.00 log CFU/g) samples, respectively. In vacuum storage, pH values remained stable at both 4 and 12 °C throughout storage. They were relatively lower in the end compared to initial pH, with values of 4.12 ± 0.00 and 4.13 ± 0.00 for C samples and 4.11 ± 0.01 and 4.14 ± 0.02 for O samples, respectively.

For samples inoculated with *E. coli* O157:H7, similarly to non-inoculated samples, the TVC, LAB, and lactic cocci/streptococci were the dominant microorganisms throughout spoilage at both storage conditions and temperatures. They remained stable until the end of storage, as seen in Figure 3 and Figure 4. In aerobic conditions at 4 °C, yeast populations at the end of storage were similar between CE (6.51 ± 0.03 log CFU/g) and OE (6.94 ± 0.56 log CFU/g) samples, and at 12 °C, they reached populations of 6.92 ± 0.18 log CFU/g for CE samples and 6.62 ± 0.16 log CFU/g for OE samples. The yeast/mould population at the end of storage in vacuum conditions was slightly lower compared to aerobic conditions in both temperatures (4 and 12 °C), for CE (5.49 ± 0.23 log CFU/g, 5.98 ± 0.17 log CFU/g) and OE (6.96 ± 1.25 log CFU/g, 6.00 ± 0.22 log CFU/g) samples, respectively. In general, *Enterobacteriaceae* populations were close to those of *E. coli* O157:H7 at all examined cases, as seen in Figure 3 and Figure 4, except for vacuum-stored samples at 12 °C, where their populations fluctuated, reaching populations of 1.59 ± 0.83 log CFU/g in CE samples and 2.52 ± 0.53 log CFU/g in OE samples at the end of storage. Regarding the populations of the pathogen, in both storage conditions (aerobic and vacuum) and temperatures (4 and 12 °C), growth was limited in early stages of storage, as seen in Figure 3 and Figure 4, and no differences were evident between CE and OE samples. The pH values on aerobic and vacuum conditions at 4 and 12 °C were similar across CE and OE samples and remained stable throughout storage. Overall, the results show that the incorporation of oregano EO in Na-alginate films did not notably influence spoilage microbiota or *E. coli* O157:H7 survival in Feta cheese stored under either aerobic or vacuum conditions, at all storage temperatures.

With regard to the samples inoculated with *L. monocytogenes*, the growth of TVC, LAB, and lactic cocci/streptococci followed similar patterns in both storage conditions and temperatures to those of non-inoculated samples, as seen in Figure 5 and Figure 6. Under aerobic storage, populations of yeast/moulds increased toward the end of storage, reaching 7.24 ± 0.12 log CFU/g and 6.66 ± 0.05 log CFU/g for CL samples, and 6.76 ± 0.05 log CFU/g and 6.63 ± 0.13 log CFU/g for OL samples, at 4 and 12 °C, respectively. However, no clear inhibitory effect of oregano EO was evident between CL and OL sample treatments regarding suppression of yeast/moulds spoilage. Similarly, at vacuum conditions, no effect of oregano EO was detected on yeast/moulds spoilage, and at the end of storage at both temperatures (4 and 12 °C), populations of yeast/moulds were slightly higher for CL (5.60 ± 0.11 log CFU/g, 5.45 ± 0.41 log CFU/g) and OL (5.86 ± 0.21 log CFU/g, 5.47 ± 0.32 log CFU/g) samples, respectively. *Enterobacteriaceae* were not detected in any treatment under any storage condition. Regarding *L. monocytogenes* populations, a reduction over time was observed under both aerobic and vacuum conditions (Figure 5 and Figure 6). In aerobic conditions at 4 °C, *L. monocytogenes* survived until day 28 in CL samples, whereas in samples containing oregano EO (OL), the pathogen was no longer detected by day 17. At 12 °C, the pathogen had a more delayed survival in CL samples, reaching 1.64 ± 0.90 log CFU/g on day 28, while in OL samples, it was detected up until day 10. In vacuum-stored samples, *L. monocytogenes* exhibited similar behaviour between treatments at 4 °C, with both CL and OL samples maintaining detectable populations until day 25, as seen in Figure 6, showing no clear effect of oregano EO at this temperature. However, at 12 °C, the pathogen survived up to day 44 in CL samples, whereas in OL samples, it was no longer detected after day 25, suggesting a potential inhibitory action of oregano EO. Throughout all treatments and storage conditions, pH values remained stable, and no clear differences were observed between control (CL) and oregano EO (OL) samples. Overall, while the general spoilage microbiota was unaffected by the oregano EO addition in Na-alginate edible films, *L. monocytogenes* survival was reduced in certain conditions, particularly under aerobic storage at 4 °C and vacuum storage at 12 °C, indicating a modest antimicrobial effect of the oregano EO.

### 3.2. Sensory Analysis

**Figure 7:**
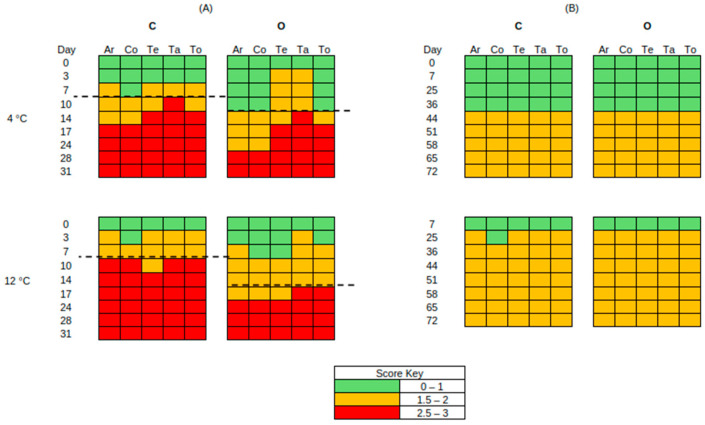
Sensory scores of aerobic (**A**) and vacuum (**B**) storage of non-inoculated Feta cheese, no-EO (C) and with EO (O) samples, stored at 4 °C and 12 °C, for aroma (Ar), colour (Co), texture (Te), taste (Ta), and total score (To). Dashed lines represent the end of shelf life.

Figure 7 shows the sensory evaluation results of non-inoculated Feta cheese samples under the tested cases. The end of shelf life was defined as the time point at which a sample was organoleptically rejected by the sensory panel. Under aerobic storage at both 4 °C and 12 °C, C samples exhibited a shorter shelf life, whereas O samples demonstrated an extension of storage time. Specifically, the shelf life of O samples was extended by 4 days at 4 °C and 7 days at 12 °C. As seen in Figure 7, across most time points, O samples received higher scores for aroma and colour and were generally preferred over C samples. At 12 °C, C samples were rejected on day 10 based on most examined attributes, while O samples maintained higher scores, indicating an important sensory improvement associated with the addition of oregano EO in Na-alginate films. In vacuum storage, sensory scores of C and O samples were comparable and showed no clear effect of oregano EO in the improvement of oranoleptic characteristics of the samples.

### 3.3. Multispectral Imaging Data

**Figure 8:**
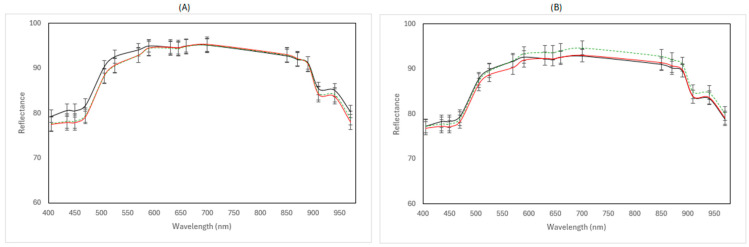
Representative multispectral imaging (MSI) reflectance spectra (mean ± standard deviation), acquired at 18 discrete wavelengths ranging from 405 to 970 nm using the benchtop-MSI instrument, corresponding to Feta cheese samples stored in aerobic (**A**) and vacuum (**B**) conditions. Fresh samples (day 0) are represented in a black solid line (──), spoiled samples at 4 °C in red line (──), and spoiled samples at 12 °C in green dashed line (----).

**Figure 9:**
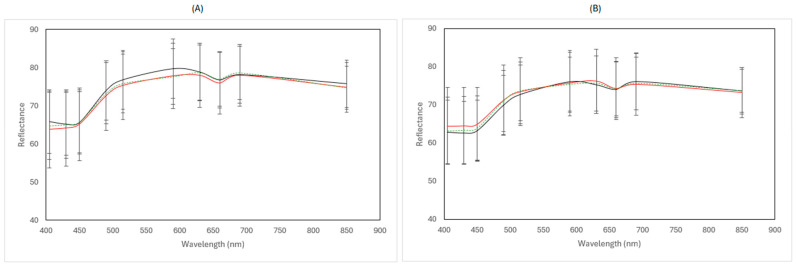
Representative multispectral imaging (MSI) reflectance spectra (mean ± standard deviation), acquired at 10 discrete wavelengths ranging from 405 to 850 nm using the portable-MSI instrument, corresponding to Feta cheese samples stored in aerobic (**A**) and vacuum (**B**) conditions. Fresh samples (day 0) are represented in a black solid line (──), spoiled samples at 4 °C in red line (──), and spoiled samples at 12 °C in green dashed line (----).

Figure 8 and Figure 9 show the representative mean spectra of fresh (day 0) and spoiled samples (end of storage) for the two MSI instruments under the different examined cases.

For the benchtop-MSI, in aerobic conditions, the reflectance of spoiled samples at both 4 and 12 °C was slightly reduced compared to fresh samples. However, there is no clear distinction between the two temperatures. In vacuum storage, while samples stored at 4 °C during the end of storage have similar reflectance values as fresh samples, at 12 °C, there is a visible increase in overall reflectance, as can be seen in Figure 8. In the cases of portable-MSI, there are no clear differences in the spectra at both aerobic and vacuum conditions, for both 4 and 12 °C.

### 3.4. Machine Learning Models

Table 2, Table 3, Table 4 and Table 5 show the performance metrics of the test set for each dataset. For aerobic storage, the R^2^ and RMSE values ranged from −0.290 to 0.726 and 0.426 to 1.345, respectively, while for vacuum storage, they ranged from −0.300 to 0.355 and 0.362 to 0.721, respectively. In most cases, the best preprocessing method for predictive performance was centering and scaling. The benchtop-MSI showed the best performance compared to portable-MSI in almost all dataset cases, as seen in Table 2, Table 3, Table 4 and Table 5. More specifically, in aerobic storage for the E. coli dataset, the best model for benchtop-MSI was the NNet (R^2^ = 0.726, RMSE = 0.426), followed by LARS (R^2^ = 0.717, RMSE = 0.428) and PLS-R (R^2^ = 0.717, RMSE = 0.431), while for portable-MSI the best model was Ridge (R^2^ = 0.361, RMSE = 0.586), followed by PLS-R (R^2^ = 0.254, RMSE = 0.642) and PCR (R^2^ = 0.249, RMSE = 0.634). In the *L. monocytogenes* dataset for benchtop-MSI, the best performance was from the LARS model (R^2^ = 0.661, RMSE = 0.696), followed by Ridge (R^2^ = 0.640, RMSE = 0.715) and SVM-Linear (R^2^ = 0.636, RMSE = 0.721), and for portable-MSI, the best model was the NNet model (R^2^ = 0.421, RMSE = 0.864), followed by Random Forest (R^2^ = 0.394, RMSE = 887) and SVM-Radial (R^2^ = 0.386, RMSE = 0.890). For vacuum storage, the overall results were not satisfactory. For the *E. coli* dataset, the best model for benchtop-MSI was the NNet (R^2^ = 0.360, RMSE = 0.429) and for portable-MSI, the NNet (R^2^ = 0.356, RMSE = 0.601). In the *L. monocytogenes* case, the best model for benchtop-MSI was the SVM-Radial (R^2^ = 0.284, RMSE = 0.362) and for portable-MSI, the kNN (R^2^ = 0.206, RMSE = 0.372). Overall, the best model performances were acquired from datasets in aerobic storage, possibly because the yeast/mould populations fluctuated more in comparison to vacuum, according to the microbiological results.

**Figure 10:**
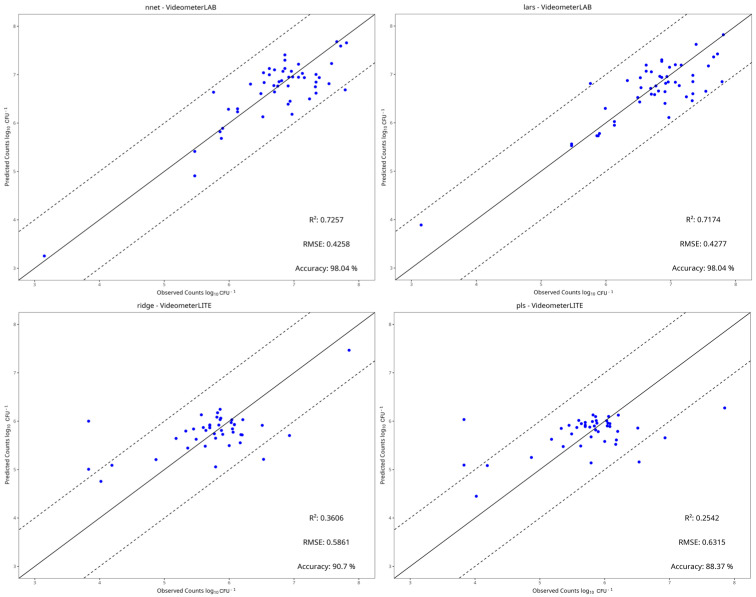
Observed versus predicted yeast/mould counts of the two best models under aerobic conditions of the *E. coli* dataset, for benchtop-MSI (**top row**) and portable-MSI (**bottom row**). The solid line represents the ideal y = x relationship, while dashed lines indicate the ±1 log CFU/g threshold of prediction accuracy.

**Figure 11:**
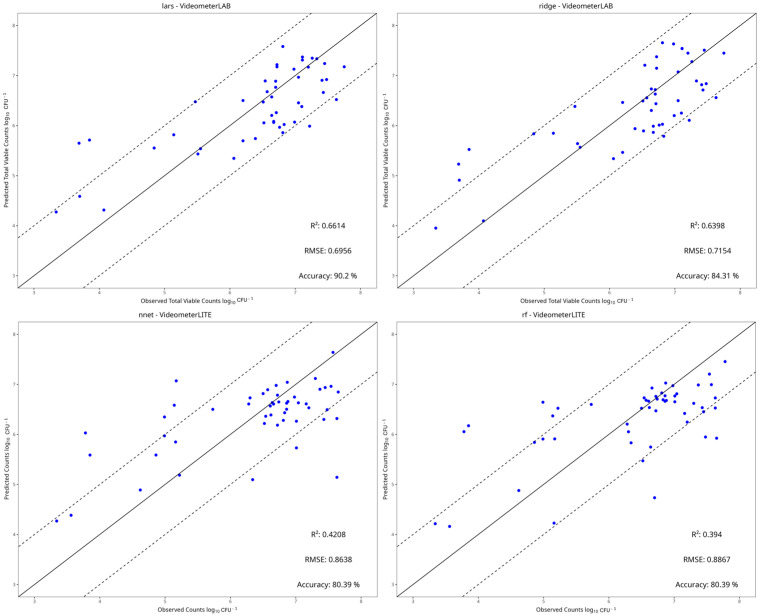
Observed versus predicted yeast/mould counts of the two best models under aerobic conditions of the *L. monocytogenes* dataset, for benchtop-MSI (**top row**) and portable-MSI (**bottom row**). The solid line represents the ideal y = x relationship, while dashed lines indicate the ±1 log CFU/g threshold of prediction accuracy.

**Figure 12:**
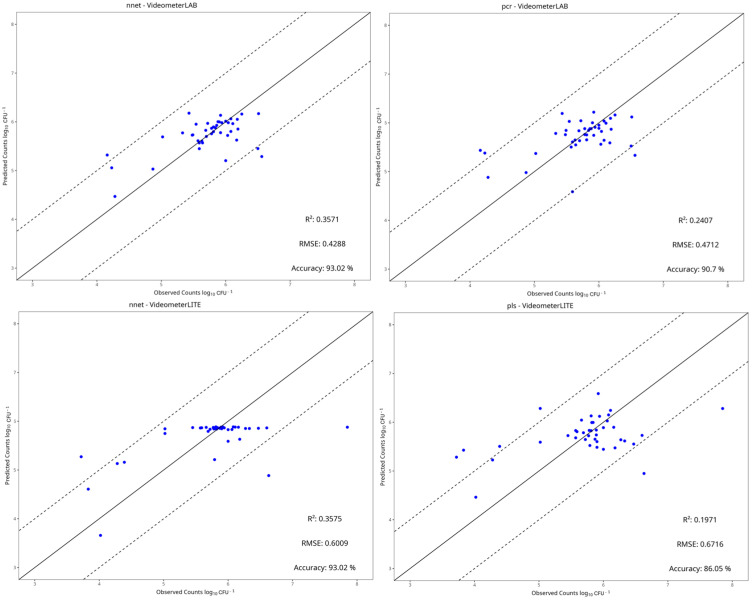
Observed versus predicted yeast/mould counts of the two best models under vacuum conditions of the *E. coli* dataset, for benchtop-MSI (**top row**) and portable-MSI (**bottom row**). The solid line represents the ideal y = x relationship, while dashed lines indicate the ±1 log CFU/g threshold of prediction accuracy.

**Figure 13:**
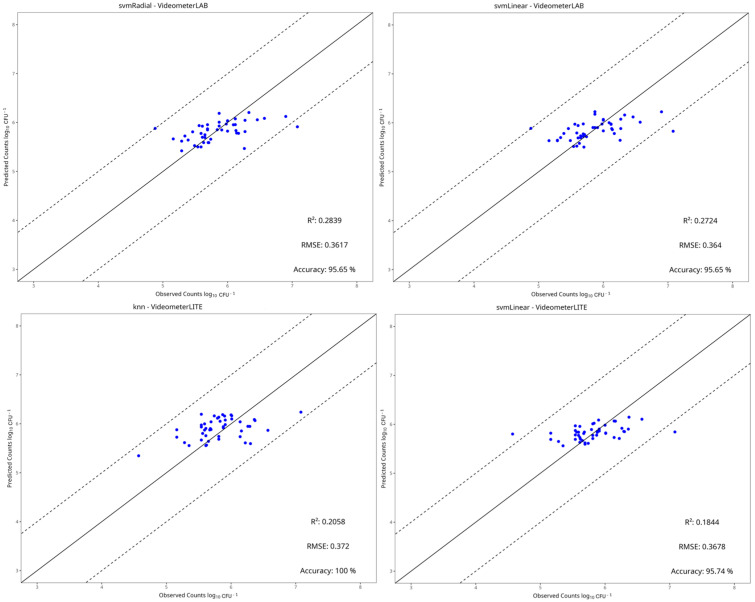
Observed versus predicted yeast/mould counts of the two best models under vacuum conditions of the *L. monocytogenes* dataset, for benchtop-MSI (**top row**) and portable-MSI (**bottom row**). The solid line represents the ideal y = x relationship, while dashed lines indicate the ±1 log CFU/g threshold of prediction accuracy.

Figure 10, Figure 11, Figure 12 and Figure 13 display the observed versus the true values of yeast/moulds of the prediction tests for the two best models in each dataset.

In aerobic condition datasets, most predictions fall within the ±1 log CFU/g range, and deviations from the ideal line are observed mostly on the portable-MSI compared to the benchtop-MSI, showing over- and underestimation of the sample’s true values. In vacuum conditions datasets, while values may fall between the ±1 log CFU/g range, a compression bias is apparent. As seen in Figure 12 and Figure 13, observed values are spread across the full measured range. However, the predicted values cluster around a narrower range, indicating a systematic tendency of the models to regress predictions toward the mean. The models tended to underestimate higher counts and overestimate lower counts, showing a stable bias.

## 4. Discussion

Recently, there has been a growing demand in smart and active packaging aimed at enhancing food quality, safety, and extension of shelf life [58]. Among these emerging technologies, edible films and coatings have achieved particular attention due to their biodegradability, ability to act as carriers of functional compounds, and the overall compatibility with environmentally sustainable strategies [13]. They can adjust the external environment and regulate the internal structure of products, therefore prolonging their shelf life [59]. Incorporating natural antimicrobials, such as essential oils, into edible films for cheese packaging has been explored as a means of inhibiting spoilage microorganisms and improving product stability without relying on synthetic additives [35,41,60].

The current study evaluated the incorporation of oregano EO into Na-alginate edible films, on the microbiological and sensory quality of Feta cheese during storage, while also assessing the effect the treatment had on the fate of two pathogenic bacteria commonly found in cheese, *L monocytogenes* and *E. coli.* The preservation of the native lactic acid bacteria (LAB) population is a critical requirement in cheese packaging applications, as they represent the dominant and most important microbial group, being primarily responsible for mechanisms such as proteolysis, lipolysis, development of characteristic sensory attributes, etc. [50,61]. Their presence in cheese microflora is essential for maintaining the identity and organoleptic quality of the product [16]. In the present study, the incorporation of oregano EO into Na-alginate edible films did not inhibit the growth of mesophilic LAB or lactic cocci, whose populations remained stable throughout storage. These results are in accordance with those reported by Marcial et al. [62], who indicated no interference of oregano EO with the native cheese microflora. Other studies have reported that the addition of EOs such as oregano, rosemary, or thyme could inhibit LAB growth or activity, potentially leading to decreased acid production and altered ripening dynamics [63]. In contrast, other research indicated no marked differences between control samples and those containing EOs, suggesting that the antimicrobial activity of these compounds may depend on their concentration and application method [64].

Yeasts and moulds are also naturally present in cheese and are considered the major spoilage microorganisms in white-brined cheeses like Feta, by causing discolouration, altered texture, off-flavours, and gas formation, which compromise product quality and shorten shelf life [9,65,66]. The use of EOs has been widely explored as a natural means to inhibit such undesirable microorganisms [66,67]. Oregano and rosemary EOs have been reported to possess strong antifungal activity in dairy products, attributed mainly to phenolic compounds such as carvacrol and thymol [61,67,68]. In a study by Said Zantar et al. [38], the addition of *Origanum compactum* EO (0.1%) to goat cheese completely inhibited the growth of yeasts and moulds, delaying the spoilage of the samples. Similarly, El-Sayed et al. [69] observed that incorporating cumin EO in nanoemulsion form into white soft cheese effectively prevented yeast and mould growth throughout storage. In contrast, other studies have shown that certain EOs, such as rosemary or myrtle, do not significantly influence mould growth during cheese ripening [16]. The limited antifungal effect observed for oregano EO under the examined conditions was reflected in the similar yeast and mould populations between control and EO cases, with only a slight delay observed in samples with oregano EO in the Na-alginate films at 4 °C, without the addition of the pathogen. Therefore, the potential of oregano EO incorporated into edible films to inhibit spoilage microorganisms in Feta cheese may depend on storage conditions, EO concentration, and the method in which it is applied to the samples.

Dairy products such as cheese are often cross-contaminated with pathogenic bacteria during different production stages, and this can not only lead to product degradation but can also pose a health hazard to consumers [18,66,70]. The most commonly encountered foodborne pathogens in such products include Gram-negative bacteria such as *Escherichia coli* O157:H7 and *Salmonella* spp., as well as Gram-positive bacteria like *Listeria monocytogenes* and *Staphylococcus aureus* [66,71]. In the present study, two major foodborne pathogens, *Listeria monocytogenes* and *Escherichia coli* O157:H7, were monitored during the storage of Feta cheese samples wrapped with Na-alginate edible films, with or without the addition of oregano EO, to assess the effect on the pathogens’ survival. In general, *E. coli* populations were low across all examined cases and followed similar trends to the total *Enterobacteriaceae* counts, with no major differences between control and EO samples. These findings indicate that the addition of oregano EO at the tested concentration did not exert an inhibitory effect on *E. coli* growth, which may be accredited to the low pH of Feta cheese, the competitive activity of LAB, and the reduced availability of nutrients [72,73,74]. Also, in another study by Motelica et al. [75], it was stated that the alginate films may be improved by the addition of ZnO nanoparticles loaded with citronella essential oil and further used on soft cheese packaging, indicating the extension of the product’s shelf life over 14 days.

In contrast, *L. monocytogenes* showed a gradual reduction in most examined conditions, although the extent depended on temperature and storage conditions. In aerobic storage, the pathogen survived longer in control samples than in EO samples at both storage temperatures, suggesting a potential inhibitory effect of oregano EO. A similar trend was observed under vacuum conditions, where EO samples showed earlier reduction in pathogen populations compared to control, especially at 12 °C. These results are consistent with former studies reporting the higher susceptibility of Gram-positive bacteria to essential oils compared to Gram-negative, possibly due to the absence of the outer lipopolysaccharide membrane that otherwise limits the diffusion of hydrophobic compounds such as carvacrol and thymol [37,68]. Similar observations were made by Govaris et al. [76], who found that oregano and thyme EOs at two different concentrations (0.1 mL/100 g and 0.2 mL/100 g) inhibited *L. monocytogenes* more effectively than *E. coli* O157:H7 in Feta cheese stored under MAP at 4 °C.

The pH of both control and EO samples remained stable throughout storage under all examined storage conditions and temperatures. This stability is consistent with the stable LAB populations observed in the present study, indicating that the acidifying activity of the native microflora was unaffected by the addition of the oregano EO. These findings agree with previous research, where the addition of EOs did not significantly impact cheese pH, while helping maintain an acid-based balance [71]. In another study on traditional Argentinian cheese, the addition of oregano EO did not alter the acidifying activity of LAB, further confirming that essential microbial fermentation processes were preserved [61].

Sensory evaluation provided a comprehensive view of how the examined treatments influenced the quality of the samples throughout storage. Under aerobic conditions, samples containing oregano EO in the Na-alginate films showed a delay in the development of spoilage-related sensory defects, such as off-odours, discolouration, and surface deterioration, compared to control samples, resulting in extended shelf life at both storage temperatures. At this point, it is worth emphasising that at the household level in Greece, the usual practice is to store feta aerobically until consumption. This favours the rapid growth of yeasts, resulting in the fast rejection of the product, as the findings of this study also highlight. In vacuum-stored samples, the examined treatments did not show notable sensory differences between the two sample groups. The sensory scores were comparable throughout storage at both storage temperatures, and the impact of oregano EO on sensory attributes and shelf-life extension was minimal. Although essential oils in dairy products may alter their sensory profile, such as distinctive taste and aroma, due to their intense volatile compounds, the concentration of oregano EO used in this study, and the fact that it was incorporated into the matrix of the Na-alginate edible films rather than applied directly to the cheese, did not negatively affect the intrinsic characteristics of Feta cheese [77,78]. This is in accordance with previous studies showing that the sensory impact of essential oils in cheese is dependent on their concentration. In fresh cheese, low oregano EO levels (0.05%) enhanced flavour and overall acceptability during storage, while higher concentrations or stronger oils such as rosemary EO showed undesirable results and early rejection by the panellists [79]. Similarly, studies on Feta cheese reported that the use of oregano or thyme EO at moderate levels (0.1–0.2 mL/100 g) was accepted and did not introduce strong herbal notes [76]. These findings align with the existing literature, demonstrating that controlled incorporation of oregano EO, particularly when released gradually through an edible film, can delay spoilage-related deterioration, especially under aerobic conditions, without affecting the natural aroma or flavour of Feta cheese. This evidence suggests that oregano EO incorporated in Na-alginate edible films offers a promising balance between antimicrobial effectiveness and sensory acceptability, contributing to the extension of shelf life in white-brined cheeses.

Despite the growing interest in spectroscopic technologies, research on applying imaging techniques to cheese for microbial evaluation, especially MSI, remains limited. To our knowledge, existing studies based on NIR, ATR-FTIR, Raman, or hyperspectral imaging predominantly focus on physicochemical properties such as fat and protein content, lipid oxidation, textural attributes, or authentication and adulteration detection [80,81,82,83]. In this study, two different MSI instruments coupled with machine learning models were evaluated for the prediction of microbial spoilage in Feta cheese by yeasts and moulds. In general, data from samples stored in aerobic conditions produced better results than those stored in vacuum packaging. The advanced yeast spoilage that was also evident through the microbiological results likely contributed to the improved predictive performance of the models. In addition, the benchtop-MSI instrument almost consistently had the most accurate results compared to the portable-MSI. This can be attributed to the instruments’ broader spectral range and higher number of wavelengths, possibly capturing subtle chemical changes associated with spoilage in cheese. Portable spectroscopic devices have gained attention in the food industry as they offer rapid, non-destructive, and in situ assessment. However, they operate with fewer wavelengths and greater sensitivity to environmental variation, factors that possibly explain their comparatively lower performance in complex matrices such as cheese [84]. Across all developed models, several algorithms demonstrated a satisfactory predictive capability for estimating the yeast and mould populations. In particular, the best models out of all cases examined were both from the benchtop-MSI. Specifically, NNet in aerobic storage data from the *E. coli* batch had an RMSE = 0.426, R^2^ = 0.726, and accuracy = 0.98, followed by LARS from data of the *L. monocytogenes* batch (RMSE = 0.696, R^2^ = 0.661, and accuracy = 0.90). Despite variability in performance across cases, Neural Networks (NNets) produced the most accurate predictions out of the other models in half of the datasets tested. Unlike conventional machine learning approaches used in food science modelling, which are mostly based on linear behaviour of variables (i.e., PLS-R), the so-called Artificial Neural Networks (ANNs), compromise computing techniques that can discover non-linear trends among variables in data, especially when these are unknown [85]. In a study by Lisak Jakopović et al. [86], the potential of ANNs for predicting brine, physical, textural, and sensory properties of white-brined cheese during storage was assessed. By using compositional and storage data as input, the models were able to accurately estimate multiple quality attributes (R^2^ up to 0.987 for brine properties and 0.87 for cheese properties). In another study, NIR spectral data were used along with ANN to predict a range of sensory properties of cheese, achieving R^2^ values between 0.73 and 0.87 [85]. These results highlight the capacity of neural networks to capture complex, non-linear relationships in multidimensional cheese datasets.

## 5. Conclusions

The application of oregano EO in Na-alginate edible films demonstrated a moderate ability to extend the shelf life of Feta cheese in aerobic and vacuum conditions. It offered a mild antimicrobial action against pathogens, more evident against *L. monocytogenes* and less against *E. coli*. Future studies could evaluate the stability and antimicrobial efficacy of EO-based coatings in cheese preservation under a wider range of storage conditions, including different EOs and concentrations that balance microbial control with sensory acceptability. The evaluation of multispectral imaging coupled with machine learning models showed a promising performance. However, further refinement of the models’ parameters is needed to improve robustness across batches, instruments, and sample conditions. Sensor fusion of integrating MSI with additional instruments may enhance prediction consistency and support real-time quality monitoring in industrial settings. Overall, the findings of the current study highlight the potential of natural preservation strategies together with advanced imaging technologies to enhance cheese safety, extend shelf life, and support modern, data-driven quality control approaches in the dairy industry.

## Figures and Tables

**Table 1:** Different sample cases and treatments of Feta cheese samples.

Storage Temperature	Packaging Conditions	Treatment		Abbreviation
4 °C/12 °C	Aerobic/Vacuum	Non- inoculated	Control edible films	C
			Edible films with oregano EO	O
		Pathogen inoculated	Control edible films with *E. coli* O157:H7 or *L. monocytogenes*	CE or CL, respectively
			Edible films with oregano EO and *E. coli* O157:H7 or *L. monocytogenes*	OE or OL, respectively

**Table 2:** Performance metrics of predictive models for yeast/moulds counts using MSI instruments under aerobic conditions of the *E. coli* dataset. The best model for each case is highlighted in bold.

Instrument	Preprocessing	Model	RMSE	MAE	R^2^	Accuracy
Benchtop-MSI	Centring and Scaling	PLS-R	0.431	0.333	0.717	0.96
		Ridge	0.447	0.360	0.681	0.96
		SVM-Linear	0.453	0.322	0.673	0.96
		SVM-Radial	0.487	0.371	0.622	0.96
		Random Forest	0.598	0.462	0.431	0.92
		kNN	0.600	0.469	0.426	0.88
		PCR	0.451	0.344	0.676	0.92
		LARS	0.428	0.327	0.717	0.98
		**NNet**	**0.426**	**0.328**	**0.726**	**0.98**
Portable-MSI	Centring and Scaling	PLS-R	0.632	0.429	0.254	0.88
		**Ridge**	**0.586**	**0.415**	**0.361**	**0.91**
		SVM-Linear	0.655	0.431	0.197	0.88
		SVM-Radial	0.707	0.436	0.063	0.86
		Random Forest	0.650	0.411	0.207	0.88
		kNN	0.716	0.449	0.039	0.86
		PCR	0.634	0.441	0.249	0.88
		LARS	0.643	0.427	0.225	0.86
		NNet	0.655	0.436	0.196	0.88

**Table 3:** Performance metrics of predictive models for yeast/moulds counts using MSI instruments under aerobic conditions of the *L. monocytogenes* dataset. The best model for each case is highlighted in bold.

Instrument	Preprocessing	Model	RMSE	MAE	R^2^	Accuracy
Benchtop-MSI	SNV	PLS-R	0.741	0.601	0.609	0.84
		Ridge	0.715	0.586	0.640	0.84
		SVM-Linear	0.721	0.571	0.636	0.88
		SVM-Radial	0.928	0.711	0.386	0.73
		Random Forest	0.903	0.665	0.419	0.69
		kNN	1.345	0.895	−0.290	0.67
		PCR	0.849	0.659	0.487	0.71
		**LARS**	**0.696**	**0.555**	**0.661**	**0.90**
		NNet	0.827	0.624	0.513	0.80
Portable-MSI	Centring and Scaling	PLS-R	1.013	0.842	0.203	0.67
		Ridge	1.039	0.849	0.162	0.69
		SVM-Linear	1.003	0.748	0.219	0.71
		SVM-Radial	0.890	0.682	0.385	0.71
		Random Forest	0.887	0.658	0.394	0.80
		kNN	0.906	0.694	0.362	0.75
		PCR	0.949	0.760	0.301	0.75
		LARS	1.028	0.839	0.179	0.69
		**NNet**	**0.864**	**0.648**	**0.421**	**0.80**

**Table 4:** Performance metrics of predictive models for yeast/moulds counts using MSI instruments under vacuum conditions of the *E. coli* dataset. The best model for each case is highlighted in bold.

Instrument	Preprocessing	Model	RMSE	MAE	R^2^	Accuracy
Benchtop-MSI	Centring and Scaling	PLS-R	0.481	0.312	0.188	0.93
		Ridge	0.609	0.346	−0.300	0.88
		SVM-Linear	0.485	0.314	0.173	0.93
		SVM-Radial	0.492	0.323	0.152	0.95
		Random Forest	0.559	0.361	−0.098	0.95
		kNN	0.562	0.392	−0.108	0.88
		PCR	0.471	0.317	0.221	0.91
		LARS	0.530	0.327	0.015	0.88
		**NNet**	**0.429**	**0.281**	**0.360**	**0.93**
Portable-MSI	None	PLS-R	0.672	0.474	0.197	0.86
		Ridge	0.684	0.510	0.165	0.84
		SVM-Linear	0.687	0.483	0.157	0.84
		SVM-Radial	0.721	0.463	0.072	0.84
		Random Forest	0.692	0.442	0.145	0.84
		kNN	0.702	0.450	0.120	0.84
		PCR	0.673	0.474	0.190	0.86
		LARS	0.687	0.463	0.155	0.86
		**NNet**	**0.601**	**0.393**	**0.356**	**0.93**

**Table 5:** Performance metrics of predictive models for yeast/moulds counts using MSI instruments under vacuum conditions of the *L. monocytogenes* dataset. The best model for each case is highlighted in bold.

Instrument	Preprocessing	Model	RMSE	MAE	R^2^	Accuracy
Benchtop-MSI	Centring and Scaling	PLS-R	0.389	0.315	0.161	0.98
		Ridge	0.452	0.340	−0.130	0.96
		SVM-Linear	0.364	0.261	0.272	0.96
		**SVM-Radial**	**0.362**	**0.257**	**0.284**	**0.96**
		Random Forest	0.389	0.293	0.161	0.96
		kNN	0.406	0.301	0.087	0.96
		PCR	0.382	0.273	0.192	0.98
		LARS	0.421	0.324	0.018	0.98
		NNet	0.381	0.286	0.195	0.98
Portable-MSI	None	PLS-R	0.390	0.293	0.074	0.96
		Ridge	0.380	0.300	0.122	0.98
		SVM-Linear	0.368	0.259	0.184	0.96
		SVM-Radial	0.389	0.285	0.079	0.96
		Random Forest	0.395	0.299	0.051	0.96
		**kNN**	**0.372**	**0.307**	**0.206**	**0.96**
		PCR	0.390	0.290	0.073	0.96
		LARS	0.394	0.289	0.056	0.96
		NNet	0.375	0.281	0.143	0.98

## Data Availability

Dataset is available on request from the authors.

## Abbreviations

The following abbreviations are used in this manuscript:

EO	Essential Oil
LAB	Lactic Acid Bacteria
TVC	Total Viable Counts
MSI	Multispectral Imaging
C	Control Samples with Na-alginate edible films
O	Samples with oregano EO in the Na-alginate edible films
CE	Control Na-alginate edible films samples inoculated with *E. coli* O157:H7
CL	Control Na-alginate edible films samples inoculated with *Listeria monocytogenes*
OE	Na-alginate edible films with oregano EO samples inoculated with *E. coli* O157:H7
OL	Na-alginate edible films with oregano EO samples inoculated with *Listeria monocytogenes*
SNV	Standard Normal Variate
PLSR	Partial Least Squares Regression
PCR	Principal Component Regression
SVM	Support Vector Machines Regression
kNN	k-Nearest Neighbours Regression
NNet	Neural Network Regression
LARs	Least Angle Regression
RMSE	Root Mean Square Error
MAE	Mean Absolute Error
ANN	Artificial Neural Networks
